# Multimorbidity in Chronic Conditions: Public Primary Care Patients in Four Greater Mekong Countries

**DOI:** 10.3390/ijerph14091019

**Published:** 2017-09-06

**Authors:** Supa Pengpid, Karl Peltzer

**Affiliations:** 1 ASEAN Institute for Health Development, Mahidol University, Salaya, Phutthamonthon, Nakhon Pathom 73170, Thailand; supaprom@yahoo.com; 2 Department of Research Innovation and Development, University of Limpopo, Sovenga 0727, South Africa; 3 HIV/AIDS/STIs and TB (HAST), Human Sciences Research Council, Pretoria 0001, South Africa

**Keywords:** chronic condition, multimorbidity, public primary care, Greater Mekong countries

## Abstract

The aim of this study was to explore the prevalence, pattern, and social determinants of chronic conditions multimorbidity among chronic disease primary care patients in four Greater Mekong countries (Cambodia, Myanmar, Thailand, and Vietnam). In a cross-sectional survey, chronic disease patients accessing primary care were recruited if they had been diagnosed with any of 21 chronic conditions in the past 12 months, and were interviewed with a structured questionnaire on anxiety, depression, alcohol use, tobacco use, dietary behaviour, physical activity, and quality of life. The sample included 6236 public primary care patients (32.8% men and 67.2% women), with a mean age of 53.0 years (SD = 16.8). From 21 chronic conditions, the three most common were hypertension (37.4%), depression (34.4%), and digestive diseases (32.0%). In all, 27.4% had one chronic condition, 28.6% had two, 22.4% had three, and 21.6% had four or more chronic conditions. The percentage with the highest comorbidity was depression (47.3%), hypertension (43.4%), and digestive diseases (34.1%). The highest mean multimorbidity reported was for mental illness (4.44), kidney disease (4.11), and Parkinson’s disease (4.10), and the lowest multimorbidity for epilepsy (2.43) and cancer (2.80). Compared to those who had only one chronic condition, being male, older age, lower education, and lower quality of life were associated with having two and three or more chronic conditions. Multimorbidity is a prevalent problem among chronic condition primary care patients—a finding with implications for health care delivery, management, and research.

## 1. Introduction 

There has been a rapid increase in non-communicable disease (NCD)-related deaths in Asian—including Greater Mekong—countries. NCDs—in particular cardiovascular disorders (CVDs), cancer, diabetes, and chronic respiratory disease—are now the major cause of death and disease burden in the region [1,2]. The epidemiological transition from communicable to NCDs has been taking place in Southeast Asia, creating a public health challenge [3]. NCDs are the number-one killer in the South Asia region, accounting for 55% of all death [4]. Globally, half of the people with NCDs have two or more conditions (multimorbidity) [5]. Multimorbidity may be defined as “the coexistence of multiple chronic conditions in a given individual” [6]. Based on the research to date [5], there seems to be no consensus on which conditions should be considered or on the method used for measuring multimorbidity [7]. Evidence from low-income settings is limited [7,8,9].

In a South African primary health care study, hypertension was the most common comorbid condition in type 2 diabetes, epilepsy, asthma, and chronic obstructive pulmonary disease (COPD), and diabetes and hypertension was the most common combination [10]. Chronic condition (hypertension, diabetes, asthma, COPD, epilepsy, and osteoarthritis) multimorbidity (two or more conditions) was present in 36.4% of patients with COPD, 23.7% with osteoarthritis, 16.3% with diabetes, 15.3% with asthma, 12.0% with hypertension, and 6.7% with epilepsy [10]. About half of the patients with chronic conditions had comorbidity, and multimorbidity was common in patients with COPD and osteoarthritis [10]. In a large primary care population with one of 24 chronic diseases aged 55 years and older in the Netherlands, the mean multimorbidity was the highest for CVD (3.39–4.97), COPD (3.40), visual disorder (3.38), rheumatoid arthritis (3.28), asthma (3.24), Parkinson’s disease (3.23), anxiety disorder (3.21), depression (3.10), osteoarthritis (3.03), and the lowest in hypertension (2.57), migraine (2.62), cancer (2.85), musculoskeletal disorder (2.95), and epilepsy (2.98) [11]. In a large primary care practice database in the United States, the prevalence of multimorbidity (two or more chronic conditions) of 24 chronic diseases was 45.2% [12]. A pattern of chronic conditions that was observed was, for example, between atherosclerosis and hypertension, coronary heart disease, diabetes mellitus, and hyperlipidaemia [12].

Factors associated with the multimorbidity of chronic conditions (two or more chronic conditions) may include older age [8,10,13,14,15], women [8,13,15], better education [14,15], higher wealth index [14,15], lower socioeconomic status [8], urban residence [14], and low quality of life [16]. There is a lack of studies investigating multimorbidity among chronic disease patients, especially in Southeast Asia. Therefore, the aim of this study was to explore the prevalence, pattern, and social determinants of chronic conditions multimorbidity among chronic disease primary care patients in four Greater Mekong countries.

## 2. Materials and Methods

### Sample and Procedure

In the study countries (Cambodia, Myanmar, Thailand, and Vietnam), a cross-sectional survey in rural and urban primary health facilities was conducted among out-patients with chronic diseases. The study facilities were convenience-selected. Each eligible patient aged ≥18 years with a chronic disease was selected from the health facility, using a systematic sampling procedure; more details can be found in [17,18].

Trained research assistants conducted interviews with the patients using structured questionnaires after obtaining informed consent. For this study, patients with one of these 21 chronic conditions were invited in the study: asthma, COPD, diabetes mellitus, hypertension, dyslipidaemia, coronary artery disease, cardiac failure, cardiac arrhythmias, stroke, arthritis, cancer, gout, Parkinson’s disease, liver disease, kidney disease, thyroid disease, stomach and intestinal diseases, epilepsy, and mental disorders [11,19]. In addition, information on four chronic conditions (daily tobacco use, alcohol use disorder, anxiety, and depression) and on dietary behaviour, physical activity, and quality of life was assessed with structured questionnaires. In Cambodia, the National Ethics Committee for Health Research, Ministry of Health, Cambodia (Reference No.: 0225NECHR); in Myanmar the Research and Ethical Committee, University of Medicine 1, Yangon; and in Vietnam the Committee for Research Ethics, Hanoi School of Public Health; and in Thailand, the Committee for Research Ethics in Social Sciences, Mahidol University (COA. No.: 2014/193.0807), approved the study protocol.

## 3. Measures

Tobacco use was assessed with four questions: “(1) Do you currently smoke any tobacco products, such as cigarettes, cigars or pipes?” If they answered yes, they were asked “(2) Do you currently smoke tobacco products daily? (3) Do you currently use any smokeless tobacco, such as snuff, chewing tobacco, betel?” If they answered yes, they were asked “(4) Do you currently use smokeless tobacco products daily?” [20].

Alcohol use disorder was assessed using the Alcohol Use Disorder Identification Test (AUDIT)-C with a cut-off score of four defined as problem drinking [21] (Cronbach alpha 0.81).

The Hospital Anxiety and Depression Scale (HADS) [22] was used to assess anxiety (seven items) and depression (seven items). A score of ≥11 was used to define anxiety and depression [22] (Cronbach alpha was 0.73 and 0.71 for anxiety and for depression, respectively).

Dietary behaviour was assessed with seven items: consumption of fast food, fruits and vegetables, sweet drinks, protein, chips and crackers, desserts and fats during the previous 7 days [23]. From these seven items, we calculated a total dietary score of up to 14 points with 0 points signifying the best dietary habits and 14 signifying the poorest dietary habits [23].

Physical activity was assessed using the General Physical Activity Questionnaire (GPAQ) [24]. The total physical activity score was used to divide participants into inactive and moderately or highly active [24].

Quality of life was assessed with the World Health Organization Quality of Life (WHOQol)-8, consisting of eight items that were derived from the WHOQOL-Bref [25]. The eight-item index consists of two items from each domain of the original WHOQOL-BREF (physical, psychological, environmental, and social) [26], and showed acceptable cross-cultural performance and a satisfactory discriminant validity [26]. Results from the two-item sub-scales and the eight-item index were summed to get sub-scale and overall WHOQoL scores, which was then transformed to a 0–100 scale, which was grouped into low, medium, and high QoL (Cronbach alpha 0.88).

Sociodemographic data obtained from all participants were age, sex, country, formal education level, marital status, and residency status.

## 4. Data Analysis

Data were analysed using the IBM Statistical Package for the Social Sciences (SPSS) for Windows version 24.0 (SPSS, Inc., Chicago, IL, USA). The proportions of chronic diseases were calculated as percentages, means, and standard deviations. Multinomial logistic regression was used by comparing two chronic conditions and three or more chronic conditions multimorbidity prevalence with having only one chronic condition (reference category). Predictor variables included sociodemographic factors, quality of life, and behavioural risk factors (physical inactivity and poor diet). Probability below 0.05 was regarded as statistically significant.

## 5. Results

The total sample included 6236 public primary care patients; 67.2% were female, 1594 from Cambodia, 1533 from Myanmar, 1549 from Thailand, and 1560 from Vietnam. The mean age of the study population was 53.0 years (SD = 16.8), ranging 18 to 94 years. More than half (59.8%) were 50 years and older, 62.0% had six or more years of education, and 45.9% were residing in urban areas. Almost half of the participants (43.1%) reported a low quality of life, 29.4% were physically inactive, and 39.3% had a poor dietary behaviour.

From 17 health record-assessed chronic conditions (coronary artery disease, cardiac failure, cardiac arrhythmias and stroke were combined into CVD) and four interview-based assessed chronic conditions (depression, anxiety, alcohol use disorder, and daily tobacco use), the most common was hypertension (37.4%), followed by depression (34.4%), digestive diseases (32.0%), anxiety (19.4%), musculoskeletal diseases (16.4%), type 2 diabetes (17.2%), daily tobacco use (16.0%), CVD (15.9%), and arthritis (15.9%). In all, 27.4% had one chronic condition, 28.6% had two, 22.4% had three, and 21.6% had four or more chronic conditions. The percentage with the highest comorbidity was depression (47.3%), followed by hypertension (43.4%), digestive diseases (34.1%), anxiety (26.6%), daily tobacco use (22.0%), CVD (20.0%), type 2 diabetes (18.9%), arthritis (18.3%), and musculoskeletal diseases (18.1%). The highest mean multimorbidity reported was for mental illness (4.44), followed by kidney disease (4.11), Parkinson’s disease (4.10), anxiety (3.74), daily tobacco use (3.62), migraine (3.62), depression (3.58), CVD (3.57), and COPD (3.56), and the lowest multimorbidity was found for epilepsy (2.43), followed by cancer (2.80) and asthma (2.82). Comparing the younger (18–49 years) with the older age group (50–94 years), in most chronic conditions the mean multimorbidity was higher among the older than younger age group, except for a few conditions: CVD, COPD, mental illness, cancer, and Parkinson’s disease (see Table 1).

Table 2 describes the top ten chronic conditions that were comorbid with six major chronic conditions. Of those who had hypertension, the highest comorbidity was with depression (34.6%), type 2 diabetes (24.8%), CVD (20.6%), and dyslipidaemia (19.0%). Hypertension had the highest comorbidity with type 2 diabetes (56.7%), CVD (48.5%), and musculoskeletal diseases (29.4%). The major comorbid chronic conditions with COPD were depression (56.9%), digestive diseases (36.2%), and daily tobacco use (31.1%) (see Table 2).

Compared to those who had only one chronic condition, being male, older age, lower education, and lower quality of life were associated with having two and three or more chronic conditions. In addition, rural residence and poor dietary behaviour were associated with three or more chronic conditions (see Table 3).

## 6. Discussion

The study found that among chronic condition primary care patients in four Greater Mekong countries, the majority (72.6%) had multimorbidity (as also found in previous studies [5,11,12]), and even higher than in South Africa [10]. This study found that hypertension was the most common comorbid condition in type 2 diabetes, COPD, CVD, and musculoskeletal diseases, while a previous study in South Africa found that it was the most common comorbid condition in type 2 diabetes, epilepsy, asthma, and COPD [10]. Similar to a previous study among primary care patients in the Netherlands with 24 chronic conditions [11], this study found that the highest mean multimorbidity was among patients with mental disorders and CVD and lowest for cancer, epilepsy, and hypertension patients.

As reviewed previously [27,28,29], in this study depression was a very common comorbid condition of type 2 diabetes, CVD, COPD, and arthritis, and anxiety was a common comorbid condition in CVD. This finding has also been confirmed in a systematic review [30] where “depression was found to be the disease that was most commonly clustered, and was paired with 8 different diseases.” Further, as also found in the systematic review [30], this study found that hypertension was the second most clustered disease with type 2 diabetes, CVD, and musculoskeletal diseases, while this was only partially true for type 2 diabetes which only highly clustered with hypertension. A common chronic conditions pattern included hypertension, type 2 diabetes, CVD, and dyslipidaemia, which was similarly observed among US American primary care patients [12]. Some of the most common chronic condition comorbidity patterns may be explained by having a common pathophysiology [31].

In agreement with previous studies [8,10,13,14,15], this study found that older chronic condition patients were more likely to have chronic condition multimorbidity than younger patients, which is consistent with the idea that additional life years add additional opportunities to develop other chronic conditions [8]. Contrary to some previous studies [8,13,15], this study found an association between male gender and chronic condition multimorbidity. The higher prevalence of chronic condition multimorbidity among men in this sample may be because alcohol and tobacco use disorders were included in the 21 chronic conditions assessed. These two substance use disorders were much more common in men than women in this study (analysis not shown). In this study, the likelihood of chronic condition multimorbidity was higher in those with lower education. This finding was also found in some other studies [8,13], while in a study in India and Indonesia [14,15], better education was associated with chronic condition multimorbidity. This study did not find an association between income or economic status and chronic condition multimorbidity, while other studies found mixed results [8,14,15].

This study may confirm the existence of an inverse association between chronic condition multimorbidity and quality of life, as found in a previous review [16]. The possible association between chronic condition multimorbidity and quality of life may have additional health care management implications in the study countries [9]. In a recent review [32], health-related quality of life in older adults with multimorbidity was ranked as the most important research topic to improve their health and health care.

This study has several limitations. This was a cross-sectional study, so causal conclusions cannot be drawn. The investigation was conducted among chronic patients from conveniently selected health facilities in the study countries, and inclusion of other health facilities could have resulted in different findings. The preselection of chronic disease patients in this study design prevented us from reporting the overall prevalence of multimorbidity among out-patients, in general resulting in a probably higher prevalence of multimorbidity among chronic disease patients. A further limitation was that the rate of health utilization among the different chronic condition patients was not assessed. This study used a count of chronic conditions as a measure of multimorbidity, implying that each chronic condition has an equal impact on an individual, while in reality disease severity, the specific combination of chronic conditions, and access to health care may affect multimorbidity.

## 7. Conclusions 

This study from public primary health care providers found a high chronic condition multimorbidity among chronic disease patients in four Greater Mekong countries. Common patterns of chronic condition multimorbidity—e.g., hypertension was strongly comorbid with depression, type 2 diabetes, CVD, and dyslipidaemia, and CVD was strongly comorbid with hypertension and depression—were identified that could help in the development of chronic condition multimorbidity guidelines and management plans in this setting. Older male patients of lower education in a rural area seem to be at the highest risk of multimorbidity, particularly in the presence of a poor diet.

## Figures and Tables

**Table 1:** Chronic condition morbidity pattern in the sample population.

Chronic Condition	%	Percentage with Comorbidity	Mean Multimorbidity (SD)-Total	Mean Multimorbidity (SD)-18–49 Years	Mean Multimorbidity (SD)-50–94 Years
Hypertension	37.4	43.5	2.98 (1.5)	2.83 (1.6)	3.01 (1.5)
Depression	34.4	47.3	3.58 (1.3)	3.39 (1.3)	3.70 (1.3)
Digestive diseases	32.0	34.1	2.99 (1.7)	2.59 (1.6)	3.59 (1.7)
Anxiety	19.4	26.6	3.74 (1.3)	3.51 (1.3)	3.88 (1.3)
Musculoskeletal diseases	16.4	18.1	3.04 (1.7)	2.84 (1.8)	3.14 (1.7)
Type 2 diabetes	16.4	18.9	3.02 (1.5)	2.86 (1.5)	3.05 (1.5)
Daily tobacco use	16.0	22.0	3.62 (1.4)	3.45 (1.4)	3.70 (1.7)
CVD ^1^	15.9	20.0	3.57 (1.7)	3.83 (1.9)	3.47 (1.6)
Arthritis	15.9	18.3	3.54 (1.8)	3.26 (1.9)	3.30 (1.6)
Dyslipidaemia	10.4	13.8	3.46 (1.4)	3.06 (1.4)	3.64 (1.4)
Alcohol use disorder	7.8	10.6	3.44 (1.3)	3.28 (1.3)	3.73 (1.4)
Migraine or frequent headaches	7.0	8.0	3.62 (2.0)	3.33 (1.9)	4.10 (1.8)
Chronic obstructive pulmonary disease	5.6	6.9	3.56 (1.8)	3.74 (1.9)	3.48 (1.8)
Asthma	4.5	4.5	2.82 (1.9)	2.52 (1.9)	3.07 (1.9)
Kidney disease	4.5	5.4	4.11 (1.9)	3.92 (1.9)	3.99 (1.7)
Liver disease	4.1	4.8	3.07 (1.7)	2.77 (1.7)	3.46 (1.7)
Mental illness	2.4	3.0	4.44 (2.2)	4.55 (2.3)	4.32 (2.0)
Thyroid disease	1.8	2.0	3.03 (1.8)	2.59 (1.6)	3.38 (1.7)
Cancer	1.5	1.6	2.80 (1.6)	2.93 (1.7)	2.74 (1.5)
Parkinson’s disease	1.6	1.8	4.10 (2.6)	5.30 (2.6)	3.45 (2.3)
Epilepsy	0.4	0.3	2.43 (1.9)	2.40 (2.0)	2.67 (0.6)

^1^ Coronary artery diseases (186, 3.8%), cardiac failure (370, 7.5%), cardiac arrhythmias (144, 2.9%), stroke (182, 3.7%). CVD: cardiovascular disease.

**Table 2:** The top ten chronic conditions that were comorbid with each chronic condition. COPD: chronic obstructive pulmonary disease.

Hypertension *N* = 2331		Type 2 Diabetes *N* = 1022		CVD *N* = 992		COPD *N* = 351		Arthritis *N* = 991		Musculoskeletal Diseases *N* = 1025	
Comorbid Condition	%		%		%		%		%		%
Depression	34.6	Hypertension	56.7	Hypertension	48.5	Depression	56.9	Depression	47.5	Hypertension	29.4
Type 2 diabetes	24.8	Depression	34.3	Depression	47.6	Digestive diseases	36.2	Digestive diseases	40.0	Digestive diseases	26.0
CVD	20.6	Anxiety	20.7	Digestive diseases	26.2	Daily tobacco use	31.1	Hypertension	28.5	Depression	24.4
Dyslipidaemia	19.0	Dyslipidaemia	19.5	Anxiety	23.2	CVD	20.8	Musculoskeletal diseases	22.0	Arthritis	21.3
Anxiety	18.9	Daily tobacco use	13.1	Arthritis	18.4	Anxiety	20.6	Anxiety	20.8	Daily tobacco use	17.1
Daily tobacco use	16.9	CVD	11.3	Daily tobacco use	17.9	Hypertension	19.4	CVD	18.5	Anxiety	16.5
Digestive diseases	14.6	Digestive diseases	12.1	Musculoskeletal diseases	13.3	Arthritis	17.9	Daily tobacco use	18.4	CVD	12.9
Musculoskeletal diseases	12.9	Arthritis	7.5	Dyslipidaemia	12.2	Musculoskeletal diseases	12.3	Migraine	12.2	Alcohol use disorder	10.0
Arthritis	12.1	Musculoskeletal diseases	7.5	Type 2 diabetes	11.6	Migraine	8.3	Kidney disease	8.4	Dyslipidaemia	9.5
Alcohol use disorder	5.3	Alcohol use disorder	3.8	Migraine	9.1	Asthma	7.4	Type 2 diabetes	7.8	Migraine	9.4

**Table 3:** Multinomial logistic regression model examining correlates of multimorbidity.

Variable	Two Chronic Conditions versus One Chronic Condition	Three or More Chronic Conditions versus One Chronic Condition
	AOR (95% CI)	AOR (95% CI)
Sex		
Female	1 (Reference)	1 (Reference)
Male	1.80 (1.43, 2.26) ***	2.19 (1.77, 2.71) ***
Age (in years)		
18–49	1 (Reference)	1 (Reference)
50–94	1.36 (1.11, 1.67) **	1.83 (1.52, 2.21) ***
Education		
Grade 0–5	1 (Reference)	1 (Reference)
Grade 6–11	0.78 (0.62, 0.99) *	0.44 (0.36, 0.54) ***
Grade 12 or more	0.59 (0.44, 0.78) ***	0.30 (0.23, 0.39) ***
Income		
Low	1 (Reference)	1 (Reference)
High	1.05 (0.85, 1.30)	0.97 (0.80, 1.18)
Geolocality		
Urban	1 (Reference)	1 (Reference)
Rural	1.06 (0.86, 1.32)	1.24 (1.02, 1.51) *
Quality of Life		
Low	1 (Reference)	1 (Reference)
Medium	0.69 (0.50, 0.96) *	0.47 (0.34, 0.64) ***
High	0.77 (0.62, 0.96) *	0.65 (0.54, 0.79) ***
Physical inactivity		
No	1 (Reference)	1 (Reference)
Yes	0.90 (0.73, 1.13)	1.20 (0.98, 1.46)
Poor diet index (scale)	1.07 (0.88, 1.31)	1.26 (1.05, 1.52) *

*** *p* < 0.001; ** *p* < 0.01; * *p* < 0.05; AOR = Adjusted Odds Ratio; CI = Confidence Interval.
